# Can AnatomicalTerms.info with its synonyms and succinct open definitions be a solution to address variations in usage of anatomical terminology?

**DOI:** 10.1007/s12565-024-00761-x

**Published:** 2024-03-23

**Authors:** O. Paul Gobée, Sara Sulaiman, Noel T. Boaz, Amy Lovejoy Mork, Ian Whitmore

**Affiliations:** 1 Department of Anatomy and Embryology, Leiden University Medical Center, Leiden, The Netherlands; 2 School of Medicine, Royal College of Surgeons in Ireland Medical University of Bahrain, Busaiteen, Kingdom of Bahrain; 3 Laboratory of Biological Anthropology and Anatomy, Integrative Centers for Science and Medicine and Virginia Museum of Natural History, Starling Avenue, Martinsville, VA 24112 USA; 4 Department of Pathology and Anatomy, Morehouse School of Medicine, Atlanta, GA 30310 USA; 5 Division of Clinical Anatomy, Department of Surgery, Stanford University School of Medicine, Stanford, CA 94305 USA; 6 Department of Anatomy and Embryology, Leiden University Medical Center, 9600, 2300 RC Leiden, The Netherlands

**Keywords:** Anatomy, Education, medical, undergraduate, Dictionaries, medical as topic, Systematized nomenclature of medicine, Open Access Publishing

## Abstract

Anatomy, the study of human structure, is foundational to medicine. Its language has a long history, with contributions from authors hailing from diverse cultures and countries, adhering to various scientific traditions, speaking different languages, and practicing medicine across a wide gamut of specialties. The resultant disparity in terms provides challenges both for students in learning and for interdisciplinary communication. We report here on a user-friendly look-up web site, “AnatomicalTerms.info” that links a *Terminologica Anatomica* term to alternative terms in usage: synonyms, polysemes, eponyms, homonyms, and terms in other languages. Accompanying open-source definitions are generated with the help of “Definition Machine” software, that supports creating the most concise and accessible definitions for anatomical terms, eschewing superfluous description, thus reducing cognitive load of learners of anatomy looking up terms. AnatomicalTerms.info is a readily accessible online source for both the authoritative and alternatively used terms that can accurately cross-reference and/or disambiguate anatomical structures across disciplinary and cultural divides. As such, it can serve as a useful educational and clinical resource that is also flexibly open to additions and expansion as anatomical and clinical needs dictate.

## Challenges in anatomical terminology

The more than 2,000-year-old history of anatomical nomenclature can be viewed as a battleground of terms supported or opposed at various times by legions of anatomists, students, and medical personnel (Kachlik et al. 2008). As new educational programs are established worldwide, local anatomical traditions may add to the divergence in the usage of terms. There are several sources of terminological variation that can potentially create confusion and miscommunication and diminish effective communication across disciplinary and geographical boundaries. The variation disadvantages students especially who are unaware of the synonyms, thus creating unnecessary extra hurdles in understanding anatomy. We categorize below the general types of challenges in anatomical terminology.

### Synonymy

Synonyms (Greek for “same name”) in anatomy are multiple terms for the same structure, creating ambiguity. Synonyms can arise from eponyms, discipline- or specialty-bound terminology, officially renamed anatomical structures where previous terms linger, clinical need for identifying yet unnamed (parts of) structures, regional and cultural differences, and colloquial terms.

An eponym is a type of synonym for an anatomical structure that is named after a person. Several structures have an eponymous term in addition to the official descriptive term. Physicians and surgeons are used to using eponyms in descriptions of diseases and clinical procedures (Taylor 2017), but anatomical eponyms are frowned upon by anatomists because they convey no descriptive information about a structure’s location in the body or its other identifying characteristics. Moreover, they often do not refer to the first person who described them; also, there can be concerns about eponymously memorializing the name of an individual who may be controversial (see below). Nevertheless, eponyms remain in common use, especially amongst clinicians. Possible reasons may be that they are felt to honor important and influential figures in the history of anatomy and medicine, or that they are felt to be more terse and immediately identifiable in daily practice. However, a proliferation of eponyms can be a recipe for confusion in anatomy. For instance, the “ileocecal valve” is also variously known as the valve of Tulp (Di Matteo et al. 2016), valve of MacAlister (Olry 2014), valve of Falloppio (Vercelli 2023), valve of Morgagni (Buttner et al. 2021), valve of Bauhin (Kutia et al. 2019), and valve of Variolo (Lierse 1987; Olry 2014).

Discipline- or specialty-bound terminology can also create confusion when used outside a specific discipline. For example, surgeons may refer to the part of the pancreatic duct in the pancreatic head as the “proximal pancreatic duct” whereas radiologists may name this part the “distal pancreatic duct” and vice-versa for the duct in the pancreatic tail (Khara et al. 2018). Officially sanctioned terms for structures sometimes become supplanted by idiosyncratic terms favored by a subset of specialists or practitioners in a specific discipline.

Renaming anatomical structures, despite with good intent to improve or clarify terminology, also contributes to part of the body of synonyms as previous terms often linger for generations. For example, the term “common bile duct” is quoted by Eycleshymer et al.’s (1917) work on the 1895 Basle Nomina Anatomica, but was simplified to “bile duct” in later terminologies. However, the previous term is still widely used, as is shown from a Pubmed search where over 18,000 articles with the term “common bile duct” have appeared since the publication of Terminologia Anatomica (TA) (https://pubmed.ncbi.nlm.nih.gov/?term=common+bile+duct&filter=years.1998-2024).

Parts of structures distinguished in clinical practice or discovered as “new” structures may not have been granted as yet separate names in the official anatomical terminology. For instance, “common femoral artery” and “superficial femoral artery” are terms used in clinical practice to distinguish between the sections of the femoral artery proximal and distal to the branching of the deep femoral artery (Benninger 2014). This results in the confusing situation that “superficial femoral artery” and “femoral artery” refer to the same blood vessel, and similarly for the “common femoral artery.”

Regional and cultural differences also influence the used anatomical terminology. For example, the iliofemoral ligament is commonly known as Bertin’s ligament in France after Exupere Joseph Bertin (1712–1781) and known as Bigelow’s ligament in the UK and the USA after Henry Jacob Bigelow (1819–1890) (Malenfant et al. 2011; Pećina 2021). Another example is the proximal deep inguinal lymph node which is known as Cloquet’s node in France and the UK after the French anatomist and surgeon Jules Germain Cloquet (1790–1883) (Loukas et al. 2007), but it is known as Rosenmüller–Lymphknoten in German-speaking countries (Buttner et al. 2021). Even in regular, non-eponymous terminology regional differences arise. English-speaking countries, for example, usually omit the word “muscle” in muscle names, sometimes leading to lack of clarity about the intended type of structure.

Colloquial terms can provide clinicians and anatomists with non-technical terms with which they can communicate effectively with patients, students, and the lay public. For example, the lay public uses terms such as armpit, arm, and collarbone whereas anatomists refer to the axilla, upper limb, and clavicle, respectively. However, it may be unclear which structures are or are not included in the colloquial term. Other sources of lack of clarity are terms used in daily parlance that are shortened to “cut corners,” e.g. “jugular vein” used instead of specifying the internal or external jugular vein.

### Polysemy

Polysemy (Greek for “many meanings”) refers to a term that can connote different structures. This type of terminological confusion is the converse of synonymy. Rather than one structure with multiple terms, polysemy is one term referencing multiple structures. Causes of polysemy are linguistic evolution or regional differences.

Divergence in meaning can arise via normal linguistic evolution or by misapplication of terms that become ensconced over time. For example, Calot’s triangle (Abdalla et al. 2013) and the semilunar line (Standring 2021) have both grown to refer to different borders in present-day usage than originally described by Calot (1891) and Spieghel (1645), respectively. Also, the perineum is defined anatomically as the volume inferior to the pelvic floor (Standring 2021), but is clinically regarded only as the area between scrotum or vaginal opening and anus (e.g. Sagnic et al. 2023).

Divergence in meaning can also arise due to differences between usage in different countries and languages. For example, the concept of anatomical “region” tends to be interpreted as a three-dimensional anatomical volume in English-speaking countries, but as a predominantly two-dimensional area of surface anatomy in mainland Europe, as discussed in FCAT meetings (1989–1998) (Whitmore, personal observation). Also, usage of the term “fascia” has been broadly interpreted by anglophone anatomists as both superficial and deep sheaths or layers of connective tissue in the body, but in mainland Europe preferentially as a term referring more narrowly to the connective tissue layer ensheathing muscles (Singer 1935; Neumann 2023).

### Homonymy

Homonyms in anatomical terminology are terms for structures which have the same spelling or pronunciation, but different meanings. For example, the term “peroneal,” referring to the lateral leg area, is frequently pronounced the same as (and can be confused with) “perineal,” referring to the part of the pelvis below the levator ani muscle (Chmielewski and Strzelec 2019). An example from personal clinical experience of a colleague of one of the authors illustrates the very real effects of this homonymous confusion. An attending physician instructed a student to examine a patient with a foot drop and especially pay attention to the “peroneal nerve”, as the patient suffered diabetes. Shortly thereafter the physician found the patient undressed in the lithotomy position on a gynecologic examination chair as the student intended to examine the “perineal” region with a resident. To avoid this type of confusion the Terminologia Anatomica, first edition (TA1) favored the terms “common fibular nerve” and “superficial fibular nerve” instead of “common peroneal nerve” and “superficial peroneal nerve” in 1998 but the former terms are persisting to the present day among many clinicians (Pitarini et al. 2022).

Other anatomical homonyms are still fully with us. “Os,” for example, can mean either “bone” or “mouth” in Latin. “Cervix” can refer to either the neck (“collum” in Terminologia Anatomica, second edition (TA2)) or to a part of the uterus (cervix uteri). “Ileum,” the distal small intestine, is pronounced the same as “ilium,” a bone of the pelvis. The anatomical malapropism, “ilioc(a)ecal junction,” rather than “ileoc(a)ecal junction” (Bogers and Van Marck 1993), is a frequently seen result of this homonymous confusion.

### Inaccuracy in literal meaning

Inaccuracy in the literal meaning of anatomical terms is another reputed cause for confusion and a reason put forward for terms to be changed. Many terms are literally accurate descriptions of a structure’s location, position, or other characteristic. The gallbladder (vesica biliaris), for example, a term meaning “bag of bile,” is still an apt term for this structure. But for other terms their literal meanings are not accurate. In the adult the left atrium is clearly not “left” but “posterior;” the right ventricle not “right,” but “anterior.” Evolving understanding may also lead, over time, to a term being regarded as inaccurate. For instance, the seminal gland is now known to secrete a fluid that is a significant component of seminal fluid and not to store sperm. Therefore, the TA has favored the term “seminal gland” instead of the older term “seminal vesicle.” Nevertheless, several terms remain in use that are partially or completely inaccurate. For instance, the ileocecal valve is probably mistermed because the ileocecal junction contains a sphincter and not a functioning valve (Pollard et al. 2012).

Literal accuracy of anatomical terms not only aids researchers and clinicians to communicate effectively across a broad spectrum of disciplines, but also importantly facilitates learning by students. However, it is important to recognize that literal accuracy of anatomical terms is an ideal. Because anatomy is a live and active science its terminology can be expected to be at any one time in flux. Practitioners will realistically accept that a number of terms, albeit literally inaccurate, are nevertheless commonly used references or labels to identify a structure or its parts. For example, in the anatomical position the “base” of the heart is located posterosuperiorly, not inferiorly as expected, but at the present time, this is the convention by which it is described. For novices, however, misleading terms do pose a problem, because it is impossible for them to tell whether a descriptive term should be taken as an accurate description or merely as a reference or label.

### Contentious or disputed terms

Finally, terms may be contentious for different reasons. For instance, Woywodt et al. (2010) argue against the use of eponyms honoring individuals who collaborated with the National Socialists of Germany in World War II. An eponymous histological term, previously in frequent use, “Clara Cell,” is affected (Winkelmann and Noack 2010; Hildebrandt 2021). The term “pudendum” has been a focus of recent debate in anatomical terminology (Draper 2021; Zdilla 2021). TA2 demoted the term “pudendum femininum” in response to this discussion (Neumann 2021a).

Changes in anatomical terms by international professional bodies have also been controversial and causes of confusion at times. The sixth edition of *Nomina Anatomica* (NA) was published by the International Anatomical Nomenclature Committee (IANC 1989) without the approval of the IFAA and its terminological recommendations were disputed. A decade of confusion and disagreements in international anatomical terminology ensued until the publication of TA1 (Whitmore 1999) with unanimous approval. More recently, the approval of TA2, with extensive changes in Latin terminology following “regular anatomy terminology” (Neumann 2021b) has proved challenging (Chmielewski and Strzelec 2019, Chmielewski 2020, 2022, 2023, Chmielewski and Domagała 2020; Russell 2022).

### Multiplicity in terms will stay

Laitman (2009) and Gobée et al. (2011) noted that the usage of a single set of standard anatomical terms by all researchers, educators, and clinicians may well be an illusory ideal. It is difficult to change a habit. We tend to use terms because we are comfortable and more familiar with them. Natural languages are not controllable (Baud et al. 2007). Despite acknowledging that a professionally endorsed TA exists and is actively maintained for this purpose (FIPAT 2019), these authors suggest that “multiple terms [for the same structure] will always remain in parallel use.”

## History

The history of anatomical terminology began with ad hoc usage in which writers invented a name where no prior one existed to their knowledge. In 1895 the Anatomische Gesellschaft (AG) gathered these terms together and published a list, approved in Basle, the Basle Nomina Anatomica (BNA). This list was entirely in Latin. The International Federation of Associations of Anatomists (IFAA), at its creation in 1903, declared one of its main objectives “the selection of a uniform nomenclature for the anatomical sciences that would be universally adopted.” Over the next few meetings of IFAA little progress was made and the Anatomical Society of Great Britain and Ireland (ASGBI) updated the BNA in 1933 as the Birmingham Revision (BR) and in 1935 AG published the Jenenser Nomina Anatomica (JNA).

In 1936 the International Anatomical Nomenclature Committee (IANC) was created although its work was suspended until the 1950s by World War II. It published, with IFAA approval, five editions of NA between 1956 and 1983—NA1-5. IANC suggested in 1985 that it should become independent of the IFAA and published NA6 without the approval of the IFAA in 1989.

At the IFAA World Congress in Rio de Janeiro in 1989 a new committee, the Federative Committee on Anatomical Terminology (FCAT) was created. Initially it worked unsuccessfully to heal the rift with IANC before embarking on a review of the terminology, starting with NA5 as the base. In 1998 FCAT published TA1 with the unanimous approval of all the society members of IFAA (Whitmore 1999). TA1 was the first to include a complete set of English equivalent terms with those in Latin.

During the following years FCAT evolved through the Federative International Committee on Anatomical Terminology (FICAT) to become the Federative International Program on Anatomical Terminology (FIPAT). This committee is still a part of the IFAA and has updated the terminology to TA2. This edition was published online in 2019 and accepted at the IFAA World Congress in 2020 (Chmielewski 2020, 2022; Neumann 2023).

## The contribution of AnatomicalTerms.info, the process of creating definitions, and resolving terminological issues

### AnatomicalTerms.info (ATI)

To alleviate the problem of confusion about anatomical terms, the website ATI (https://anatomicalterms.info) was developed by Leiden University Medical Center together with the Clinical Anatomical Terminology (CAT) Committee of the American Association of Clinical Anatomists (AACA) (Gobée et al. 2011). It presents listings of both the IFAA official terms and other terms in use for a structure. This allows users to look-up synonyms, i.e. which terms have the same meaning, and also points users to the IFAA official term as published in TA. It is comprehensive, freely accessible, and user-friendly. To accommodate for new needs and developments it is a wiki site, meaning that it is expandable. This approach to maximally ease access to terminology through an open online presentation was previously advocated by Laitman (2009).

### The need for open and succinct definitions

To determine whether terms are correctly listed as synonyms, definitions are needed. No freely available, open-licensed definitions were available. Therefore, definitions are being created in ATI. To allow anyone access without financial barriers and to allow free reuse, definitions should be open-licensed (UNESCO 2019; Wiley 2023). Therefore, the created definitions are licensed under a Creative Commons license. Our approach has been to develop definitions of terms in standardized formats that are both uniquely defining, succinct, and accessible to all users of the terminology, meaning that they should be easily understood without an extensive anatomical background, as well as by non-native speakers of English.

Succinct and accessible definitions reduce the cognitive load for students. Gross Anatomy is generally considered far and away the most difficult course in medical school, e.g. https://strawpoll.com/most-difficult-class-medical-school. Reducing the cognitive load for students is a neuroscience-based approach to improving outcomes in medical education (Merrienboer and Sweller 2009, Leppink and van den Heuvel 2015; Szulewski et al. 2021). ATI does so by clarifying synonyms and by offering succinct and accessible anatomical definitions. To create such definitions, we try to identify the most “essential” characteristics of the structure to be defined (see below) and to preferentially use plain English terms in the definitions instead of traditional anatomical terms (e.g. “elevation” instead of “tuberosity,” “trochanter,” or “epicondyle”). For instance, ATI’s definition for the biceps brachii muscle will be “two-headed muscle contained in the anterior arm” instead of a more common, descriptive definition such as “fusiform skeletal striated muscle attached proximally with a short head to the coracoid process of the scapula and with a long head to the supraglenoid tubercle of the scapula and distally to the tuberosity of the radius and to the ulna via the bicipital aponeurosis.” Following Merrienboer and Sweller (2009) ATI’s succinct and accessible definitions decrease students’ “intrinsic load” (“direct function of the complexity of the subject matter and the expertise of the learner”) by decreasing the number of knowledge elements to be managed simultaneously in working memory to understand the definition. The details can be learned later on. This approach employs the so-called simple-to-complex learning strategy. It also reduces “extraneous load” (“superfluous processes that do not directly contribute to learning”) by preventing, so far as possible, the need to look up additional subject matter (as would probably be needed when using the descriptive definition), thus decreasing split attention and redundancy.

### Creating definitions

Members of the AACA CAT Committee manually created definitions in parallel for several structures and then abstracted common patterns from those definitions via group discussions. These patterns and accompanying guidelines were laid down in an 80-page document on “Patterns and Guidelines” for the definitions (available from the authors by request).

Using this document, cooperating anatomists started to create definitions. It soon became apparent that the written “Patterns and Guidelines” were insufficient to answer all encountered issues and reach the set goals for ATI. This was partially because of lack of coverage of different situations by the devised “Patterns and Guidelines” and partially because the goal of succinctness appeared counter-intuitive for anatomists, who tend to describe structures in detail.

Therefore, a new approach was taken: an application built on PHP general-purpose scripting language (https://www.php.net/), was created, spearheaded by one of the authors (OPG). Termed the “Definition Machine,” (DM) it is a guiding tool that encompasses the “Patterns and Guidelines” and leads an anatomist through a process, step-by-step, constructing the definition.

### Challenge of balancing the contradictory goals

When programming the DM it often appeared necessary to further refine the patterns. It proved a challenge to balance the contradictory goals of “uniquely defining,” succinctness, and accessibility. Logical, linguistic, user-friendly, anatomical, and technical aspects often conflicted.

Commonly used descriptions may be long-winded and require prior anatomical knowledge. Instead of meticulously describing a structure, we aimed to identify the “most striking characteristics” of a structure, in line with a long tradition of defining that attempts to capture the Aristotelian *essence* of a concept (Soanes and Stevenson 2003, Cimino 1998, ISO Standard 704 2009, Navarro 2014; Wikipedia: Essentialism https://en.wikipedia.org/wiki/Essentialism; Wikipedia: Definition https://en.wikipedia.org/wiki/Definition). But which characteristic or combination of characteristics of a structure, for instance a muscle, is most striking, or essential? Is it its function, location, or appearance? And how many characteristics are needed to uniquely define the structure? For instance, a definition for biceps brachii such as “two-headed muscle of the anterior arm” is succinct, but it is not yet “uniquely defining” as it may be interpreted to also cover the pectoralis major muscle. Hence, an extra characteristic must be added to ensure that only the biceps brachii is meant. Adding more characteristics of a structure to a definition assures it is “uniquely defining” but makes it less succinct. The goals “uniquely defining” and “succinct” may conflict.

There are two different purposes of definitions: identifying and explaining. The International Organization for Standardization (ISO Standard 704 2009) highlights the “identifying” function: “a definition’s main purpose is to provide enough understanding so as to avoid confusing the concept in question with other related concepts.” Other authors stress the explanatory function, e.g. “[t]he purpose of a definition is to clarify the understanding of a concept” (Navarro 2014). These two goals may conflict. For instance, the previously mentioned definition for biceps brachii as “two-headed muscle of the anterior arm” evokes a clear view in the mind of the muscle and is easily understandable, but is insufficiently discriminating from other structures, as described. To make a definition uniquely defining it may be necessary to focus on specific anatomical details, which require prior knowledge, thus making the definition less accessible.

For different types of structures (e.g. muscles with clearly two ends, versus muscles with multiple attachments, versus sphincters), different formats (patterns) of definitions in the DM are needed for the most concise definition. Traditionally used categories, such as “long bones,” “irregular bones,” “striated muscle,” or “smooth muscle” are insufficient to uniquely define specific structures, require prior knowledge of those categories, and were often felt to be superfluous to our definitions. Often it was a challenge to abstract the existing variation in muscles, bones, or vessels into categories with specific definition patterns, such that the logic and accompanying questions guiding the anatomist creating a definition, would be unequivocally understood. The DM’s abstract computer logic, requiring discrete choices, was often unnatural for anatomists who are used to the nuance and continuity in variations in biology.

A different choice of wording sometimes helped overcome conflicts between requirements. At the same time, we strived to use wording that also would be understood by non-native speakers of English. In the example of the biceps definition, we chose to make the definition “uniquely defining” by replacing the wording “*of* the anterior arm” by “*contained in* the anterior arm” as a succinct solution that still does not require additional prior knowledge and is understandable for all.

By comparing and discussing sample definitions and balancing technical, logical, linguistic, user-friendly, and anatomical aspects in the developed patterns, the group attempted to resolve the often-contradicting goals, without claiming that *the* perfect solution was found.

### Process of definition creation

**Fig. 1:**
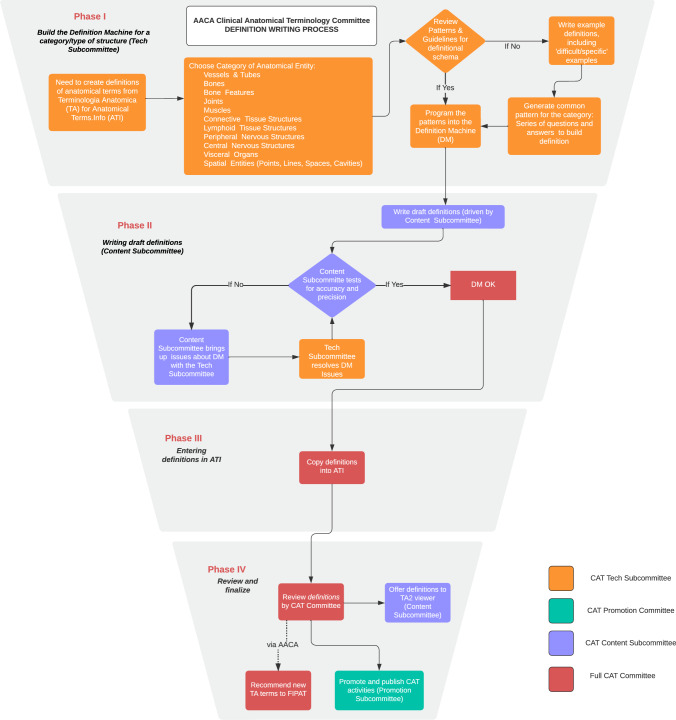
The four phases of the process of the definitional writing process for AnatomicalTerms.Info (ATI). The color-coded contributions of the three subcommittees of CAT are shown as they interacted with each other and with the CAT Committee as a whole in generating consistent, succinct, and understandable definitions of standard *Terminologia Anatomica* (TA) terminology

The final process for constructing definitions was operationalized over a period of 5 years (2018–2023) by the writers and other members of the AACA CAT Committee. The flowchart for the four phases of the process is diagrammed in Fig. 1.

The AACA CAT Committee formed three subcommittees. A Tech Subcommittee focused on Phase I, the setting up of the DM software for defining terms for the different anatomical systems and regions, such as bones, muscles, and “tubes” (vessels and ducts). This group reviewed the existing patterns and “programmed” them into the software.

A Content Subcommittee then focused on Phase II, the writing of draft definitions for all systems and regions with the help of the completed DM software for those systems and regions. Oftentimes, new unforeseen issues arose, in which case the Tech Subcommittee was consulted to solve the issue. In case of doubt, issues were discussed amongst the larger group of the CAT Committee as a whole. A Promotions Subcommittee focused on outreach of ATI and also interfaced with the CAT Committee as a whole in Phase IV. When a draft definition was completed in Phase II it was placed in ATI (Phase III) under a Creative Commons license. Finally, review and finalization of the definition took place in Phase IV.

## Conclusion

ATI is an informational resource that can effectively serve several important constituencies. It was initially created by one of our authors (OPG), based at Leiden University Medical Center. A collaboration was started in 2010 with the CAT Committee of the AACA. ATI creates a much-needed conduit between clinicians and anatomists. ATI, in an easily accessible format, can inform clinicians of the standardized (“correct”) anatomical terminology in place of favored eponyms and other “cross-cultural” synonyms, as well as inform anatomists of newly recognized or otherwise needed anatomical terms that should be added to the anatomical lexicon. Anatomy and medicine must speak the same language in order for both to advance.

Equally important is the role that ATI can play to help students in their learning. The look-up function of synonyms helps them bridge the disparity in terms they encounter. The succinct and accessible definitions reduce cognitive load in learning anatomy.

## Data Availability

The ‘Patterns and Guidelines’ document used in the generation of definitions is available by request from the first author. The definitions created up till now can be viewed on the ATI web site with the concerning terms. Access to the ‘Definition Machine’ application can be requested from the first author.
